# Psychology of nerve injury, repair, and recovery: a systematic review

**DOI:** 10.3389/fresc.2024.1421704

**Published:** 2024-11-06

**Authors:** Yaw Adu, Cameron T. Cox, Evan J. Hernandez, Christina Zhu, Zachary Trevino, Brendan J. MacKay

**Affiliations:** ^1^ School of Medicine, Texas Tech University Health Sciences Center, Lubbock, TX, United States; ^2^ Department of Orthopaedic Surgery and Rehabilitation, School of Medicine, Texas Tech University Health Science Center Lubbock, Lubbock, TX, United States; ^3^ Community, Family, and Addiction Science, Texas Tech University, Lubbock, TX, United States

**Keywords:** peripheral nerve injury (PNI), psychological factors, psychosocial, peripheral neuropathy, nerve repair, nerve recovery

## Abstract

**Background:**

Peripheral nerve injuries (PNIs) are associated with significant physical and psychological challenges, impacting both functional recovery and quality of life. Despite the physical focus of traditional treatments, psychological factors play a crucial role in the outcomes of PNI repair and recovery.

**Objectives:**

This systematic review aims to evaluate the impact of psychological and social factors on the repair and recovery of peripheral nerve injuries.

**Methods:**

A comprehensive literature search was conducted in PubMed/Medline, EMBASE, and Cochrane databases, covering studies from January 1985 to December 2022. A total of 36,190 records were identified, and after screening with Rayyan AI and applying inclusion criteria, 111 articles were selected for review.

**Results:**

The review highlights that pre-existing psychological conditions, as well as psychological responses to the injury and treatment, significantly influence recovery outcomes in PNI patients. Psychological interventions, when integrated into standard care, may improve functional recovery and quality of life.

**Conclusions:**

Psychosocial factors are critical in the management of PNIs and should be incorporated into treatment algorithms to enhance patient outcomes. Future research should focus on developing and integrating psychological support strategies in PNI treatment protocols.

## Introduction

Peripheral nerve injuries (PNIs) often result from traumatic (e.g., penetrating injury, crush, stretch, etc.) and non-traumatic (e.g., chronic compression) injuries (1, 2). While overuse is a contributing factor in certain chronic compression neuropathies, it is not considered a leading cause of all PNIs. PNIs have an estimated incidence of 13–23 per 100,000 persons each year in developed countries and can negatively impact autonomic, motor, and sensory function (3, 4). Symptomatic nerve injuries most frequently involve upper limbs with a male predominance and affect 2.4% of the general population and up to 8% of elderly patients in the United States (5, 6). Non-surgical treatment methods include medications, laser therapy, and physical therapy (7). Medications typically refer to analgesics used to manage symptoms related to PNIs, such as pain. Laser therapy, while not a primary treatment modality, may be considered in specific cases for its potential to alleviate neuropathic pain and promote nerve regeneration. In cases where conservative treatment fails, surgical options include direct suturing, use of conduits or adhesives, and autologous or allogenic nerve grafts (1, 4, 8). PNI symptoms vary greatly between patients, and untreated or unsuccessfully treated neuropathy can result in chronic pain, impaired sensation, and motor deficits.

Current literature has identified a variety of prognostic factors for PNI outcomes, including: repair method and materials, nerve injured, patient age, mechanism of injury, defect length, injury location, and time from injury to repair (9). He et al. analyzed 71 articles and found that the nerve injured was the most important factor predicting “good to excellent” nerve recovery (9). Quantitative biomedical measures (e.g., strength, range of motion, and sensory testing) and pain scores are often considered sufficient to asses PNIs both pre- and post-operatively (10, 11). Although central to predicting outcomes, these measures sometimes fall short of explaining variable outcomes in patients with seemingly similar injuries and potential for recovery.

Psychosocial factors are broadly known to play a role in both the pre- and post-treatment nerve symptoms (12, 13). Additionally, psychological issues can stem from the injury event and/or recovery process. Mason et al. identified psychiatric disorders in 43.4% of working age males (17–60 years old) 6 months after accidental injury requiring hospitalization (13). A study of adult patients treated for minor traumatic injuries showed an elevated rate of subsequent mental illnesses diagnosis, and those with diagnosed depression were more likely to have a prolonged stay in the hospital and decreased odds of return to pre-injury levels of daily activity and pre-injury work status (14). Kellezi et al. further supported these results, reporting that depression 1 month post injury is associated with decreased functional recovery (15).

In 1995, McAllister et al. hypothesized that psychological factors may account for paradoxical clinical findings post-PNI (16). A more recent study showed a significant correlation between post-traumatic stress disorder (PTSD) following traumatic upper limb PNIs and reduced function at 12 months (17). One review of 12 studies found that 32%–84% of amputees were diagnosed with psychiatric disorders post injury (18). Fortunately, the prognosis of these patients can significantly improve following psychiatric evaluation and treatment. Parashar et al. described the case of a 14-year-old male amputee who suffered from PNI induced involuntary movements, leading to anxiety and depression (19). After 2 months of psychological intervention, the patient had significant improvement of pain and dystonic symptoms (19).

Psychological factors are often modifiable, and an improved understanding of their impact on PNI recovery could lead to improved treatment algorithms in these populations. While limited data has been published, the literature lacks a comprehensive review of the evidence surrounding the pre- and post-injury impacts of psychosocial factors in PNI. We performed a systematic review to evaluate the impact of psychological and social factors on peripheral nerve injury, repair, and recovery.

## Methods

In conducting this systematic review, we followed the Preferred Reporting Items for Systematic Reviews and Meta-Analyses (PRISMA) guidelines to ensure a rigorous and transparent process (20). Our literature search was conducted using PubMed/Medline, EMBASE, and Cochrane databases, covering articles published from January 1985 through December 2022.

To identify relevant studies, we employed a set of keyword combinations (Table 1). For the initial screening of titles and abstracts, we utilized Rayyan Artificial Intelligence (AI), a web-based tool designed to facilitate the screening process by allowing multiple reviewers to work independently. Rayyan's blinding feature ensured that each reviewer's decisions were unbiased, and its machine learning algorithms helped streamline the selection process by predicting the likelihood of inclusion based on previous decisions.

**Table 1:** Terms used for literature search of pubMed/MEDLINE, EMBASE, and cochrane.

Psychological +	Nerve, Peripheral nerve, Nerve injury, Neuropathy, Iatrogenic nerve, Traumatic nerve injury, Nerve trauma, Acute nerve injury, Chronic nerve, Nerve compression, Nerve decompression, Carpal tunnel, Cubital tunnel, Dupuytren's, Pain, Sensation, Sensory nerve, peripheral nerve, nerve injury
Psychosocial +	
Psychology +	
Patient factors +	
Anxiety +	
Depression +	
Post-traumatic stress disorder (PTSD) +	

After identifying 36,190 records, we removed 3,829 duplicates, resulting in 32,361 articles for further screening. Rayyan AI was instrumental in excluding 32,066 articles based on relevance, leaving 295 articles for full-text review. These were assessed by two independent reviewers to ensure that only studies directly related to the psychological or psychosocial factors affecting peripheral nerve injury (PNI) repair and recovery were included.

**Figure 1:**
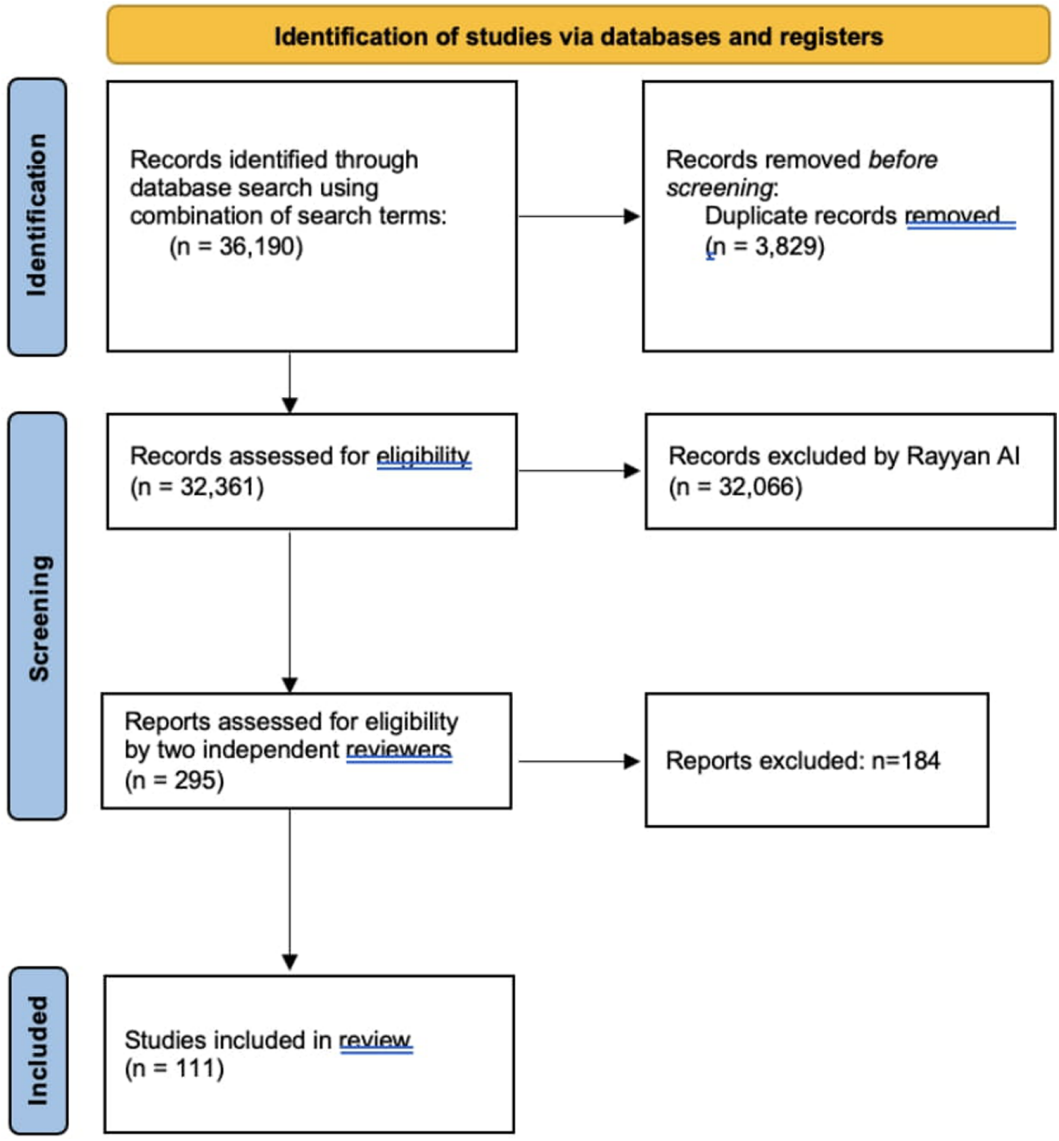
PRISMA guidelines.

Ultimately, 111 articles met our inclusion criteria and were incorporated into the synthesis of our review. While we did not use additional software like GRADE or RevMan for further quality assessment, we believe that our comprehensive approach, including the use of Rayyan AI, provided a robust and unbiased selection of studies that are directly relevant to our research question (Figure 1).

## Results

Under the broad category of PNI, there are subsets of patients and injury patterns, each with unique characteristics and challenges. In what follows, we have divided nerve injuries into acute (traumatic) and chronic subcategories and present the relevant literature with regards to each specific nerve and/or syndrome.

### Acute (traumatic) peripheral nerve injury

A database study of 16 million insured patients found 1.64% of those with extremity trauma had symptomatic PNI within 90 days (21). Patients with nerve injuries were more likely to have extended hospital stays compared to traumatic injuries without nerve involvement, despite similar rates of reported pain severity (22). A study of 158 traumatic PNIs found that patients with comorbid neuropathic pain were more likely to report lower quality of life, with a strong negative correlation between quality of life and severity of pain (23). Other studies have shown that upper extremity injuries with PNIs can result in higher prevalence of anxiety, depression, and other negative psychological symptoms (24, 25).

#### Median, ulnar, and combined traumitic nerve injuries

Ultee et al. evaluated 61 nerve surgery patients for PTSD using the Impact of Event Scale (a measure of distress after trauma). Results showed that within the first month, 92% of patients suffered from psychological stress, and 25% had scores indicating a need for psychological intervention. At 3 months, 83% showed psychological distress, and 13% had scores indicating a need for psychological intervention. Female gender and age were associated with psychological distress 1 month post-surgery, and only increased age was associated with psychological distress at 3 months (26).

Hundepool et al. conducted a study to identify prognostic factors for outcomes of median, ulnar, and combined nerve injuries in the hand (27). They found that several factors were associated with worse outcomes for hand sensibility, including older age, male gender, combined nerve injuries, and proximal nerve injuries. Younger age, higher levels of education, and lower levels of PTSD at 1 month were associated with a higher power grip. Additionally, older age and PTSD at one and three months were linked to worse Disabilities of the Arm, Shoulder and Hand (DASH) scores (27).

#### Brachial plexus injuries (BPIs)

The brachial plexus is a network of spinal nerves divided into three trunks: upper (C5-C6), middle (C7), and lower (C8-T1). These trunks are vulnerable to traumatic injuries, which can result in specific nerve palsies (28). Overextension of the neck and shoulder or penetrating injuries can lead to upper brachial plexus injuries (29). Lower brachial plexus injuries most often occur when the upper extremities are pulled upward suddenly (28–30). Estimating the incidence of brachial plexus injury is difficult due to lack of reporting, but studies show that a majority of injuries occur in males between age 15–25 and approximately 72% of BPIs occur via road traffic accidents (31, 32). Patients with BPIs had higher disability scores on the DASH questionnaire compared to those with single proximal nerve injury and distal nerve injury (33). Among all PNIs, BPIs are considered the most care-consuming, as they frequently result in long hospital stays (34).

BPIs are known to cause impairment beyond the zone of injury, often producing negative psychosocial outcomes such as poor self-image, anger, and depression (28, 35). In a study of 12 patients with BPIs, Franzblau et al. found that “sudden loss of ability” caused anger and frustration in 50% of patients immediately after injury (35). 33% of patients continued to have these same feelings throughout their recovery process due to “inability to accomplish tasks or participate in their usual activities”. To manage their illnesses, patients typically used four coping mechanisms which resulted in improvements of daily function - social and emotional support, acceptance, adaptation, and active coping (35).

Lazzeri de Medeiros et al. conducted a cross-sectional analysis to compare outcomes in children between a normal control group and a neonatal brachial plexus palsy (NBPP) group. They found that children with NBPP had significantly lower scores in happiness, mental health, behavior, and family activities. The negative psychosocial outcomes of NBPP were not limited to the children affected; their parents also exhibited significant emotional deficits on questionnaires compared to controls (36). Another study found that brachial plexus injuries at birth lead to poor mental health during adolescence and a resulting increase in psychotropic medication use (37). A study of 21 patients found that 33.3% showed signs of suicidal idealization, 19% showed symptoms of PTSD, and 19% had clinical depression with showed no changes in tobacco, alcohol, or substance abuse (38). Many patients also presented with high levels of hyperarousal, avoidance behaviors, and reexperiencing emotional and physical symptoms (38).

#### Upper and lower limb amputation

In 2005, there were approximately 1.6 million amputees in the United States, with an estimated 185,000 amputations occurring per year (39, 40). Amputation can result in various peripheral neuropathies such as phantom sensation, phantom limb pain, and neuropathic residual limb pain, all of which all can lead to negative psychosocial outcomes (41, 42). Although the mechanism behind phantom limb pain is not fully understood, it is thought to include both peripheral dysfunction and abberant cortical signalling (43). The severity of phantom limb pain is often correlated with and modulated by emotional factors (44). Patients who undergo limb salvage/reconstruction (in injuries severe enough to warrant amputation) have similar psychological outcomes at 12 and 24 months post-opertaviley (45).

Bhutani et al. conducted a study of 50 male traumatic amputees - 29 lower and 21 upper limb using the Hospital Anxiety and Depression Scale (HADS) questionnaire (46). Higher anxiety was correlated with worse outcomes in the following domains: pain perception, length of hospital stays, number of hospitalizations, number of follow ups per year, and rehabilitation satisfaction (46). Depression was only correlated with pain perception (*p* = 0.031) (46).

A study of 307 upper limb amputees found that 55.4% met criteria for clinical depression, 23.4% had diagnosable PTSD, and 20.8% were suffering from both depression and PTSD. Women and ethnic minorities were more likely to have PTSD and/or depression (47). Younger patients were more likely to have PTSD or simultaneous PTSD and depression, while older patients were more likely to have only depression or no psychological symptoms (47). Activity restriction was significantly correlated with increased rates of depression and PTSD (47). In a study of 46 traumatic upper limb amputees, 67.1% were diagnosed with depression (30.4%), PTSD (23.9%), anxiety (13.0%), panic disorder (4.3%), or adjustment disorder (23.9%) (48).

Cavanagh et al. postulated that development of PTSD may influenced by the mechanism of amputation (e.g., traumatic vs. scheduled surgical amputation) (49). Of 23 patients with amputation due to chronic conditions, only 1 developed symptoms of PTSD, and 2/3 traumatic amputees were diagnosed with PTSD. More than half of the patients with chronic amputation reported “feelings of shock, disappointment, and/or sadness”, but 10/23 also reported feelings of acceptance and relief. Traumatic amputees reported similar feelings of shock and sadness, but expressed disbelief at the loss of limb (49, 50).

A study of 12 traumatic lower limb amputations found that 75% of patients were unemployed, and 75% had expenses equal to or greater than their incomes (51). Patients experiencing these financial difficults also reported poor self-esteem and body image (51). Another study showed that lower limb amputees reported low quality of life and self-esteem, and that phantom sensations were associated with greater deficits in quality of life (52).

In a study of 110 patients, Ghous et al. found that the majority of amputees had mild depression on the Beck Depression Inventory (53). On average, amputees using assistive devices such as prosthetic limbs had lower rates of depression and less severe depressive symptoms (53).

Atherton et al. found lower levels of depression in a cohort of prosthetic wearing amputees compared to historical norms in amputees with no prosthesis (54). Patients with high public self-consciousness were more likely to suffer from psychological distress; however, private self-consciousness was not correlated with psychologic disturbances (54).

Phelps et al. found that cognitive processing was an important predictor of psychological outcomes in patients post-amputation (55). Positive cognitive processing was significantly associated with fewer depressive symptoms at 6 and 12 months and greater post-traumatic emotional growth at 12 months. Negative cognitive processing was associated with higher levels of depression and PTSD symptoms at 6 months (55).

Delehanty et al. conducted a study comparing amputees receiving psychoeducational intervention with a control group (56). The psychoeducation intervention provided information about their amputation, educated that future stressors were normal, and taught proper coping strategies. This group later reported lower levels of distress compared to controls (56).

### Chronic peripheral nerve injury

#### Sciatica

Sciatica has an annual incidence of 1%–5% and is charactarized by compression of the sciatic nerve, resulting in pain and discomfort in the lower back, buttocks, and legs (57). Mechanisms that cause the onset of sciatica are most frequently related to mechanical strain and/or poor biomechanics under physical workload, while the persistence of symptoms have been associated with psychosocial factors (58). Patients can present with or without neuropathic pain, and presence of neuropathic pain is correlated with significantly higher anxiety and depression scores (59).

Tutoglu et al. found that sciatica results in variable presentations of coping styles, defensive styles, and defensive mechanisms (59). Patients may use behavioral strategies (e.g., pacing) or cognitive strategies (e.g., distraction and calming) to help calm neuroticism associated with sciatica flares. When divided into sciatica subcategories (with or without neuropathic pain), only sciatica with neuropathic pain was independently associated with “acting out” and increased depression - measured via the Short Form-36, a quality of life questionnaire (59).

Pietri-Taleb et al. conducted a study (*n* = 1,149) assessing personality disorders and psychological distress in a cohort of white collar and blue collar workers, none of which were diagnosed with sciatica at the beginning of the study (60). At 3 years follow up, overall incidence of sciatica was 19%, with higher rates in blue collar workers. Only hysteria was significantly associated with increased sciatic pain, and only blue collar workers had significantly higher rates of hysteria (60).

Treatment modalities for sciatica vary by severity of symptoms and efficacy of interventions may be affected by psychological factors. In a prospective study of 507 sciatica patients, results showed a significant association between mental illnesses preceding treatment and prospective outcomes, with worse mental health at baseline prediciting greater symptom frequency (61). Managing expectations and education patients regarding their condition may aid in optimizing psychological factors in sciatica. It has been reported that establishing a cause of sciatica prior to offical diagnosis is beneficial for patients’ understanding and adaptive processing of their condition, and may ultimately lead to improved outcomes (62). The use of cognitive behavioral therapy in conjunction with medical treatment has also shown utility in reducing sciatica symptoms (63, 64).

#### Carpal tunnel syndrome

Carpel tunnel syndrome (CTS) is an entrapment neuropathy of the median nerve, with a prevalence of 5% in the United States and peak occurrence in ages 40–60 (65). CTS is associated with a wide variety of initiating factors, thus presentation and progression vary in individual patients (65). CTS with pain symptoms has been linked to lower quality of life compared to painless CTS (66). Psychosocial factors have been linked to higher incidence of CTS as well as poor disease progression and/or recovery from treatment (67). In a population study of 5.8 million people assessing use of psychotropic medications (psycholeptics, antidepressants, or psychoanaleptics), subjects with a diagnosis of CTS, ulnar nerve entrapment (UNE), or both were found to use psychotropic medications at higher rates than the general population (68). Current treatment methods for carpal tunnel include steroids, physical therapy, surgery, etc. (69). CTR is typically reserved for more debilitating cases, and younger age is associated with higher patient satisfaction post-operation (70). Patients undergoing CTR in the operating room have shown significantly higher levels of anxiety compared to those who received treatment in office, and patients with pre-existing diagnoses of anxiety are more likely to opt for wide awake virtual reality (WAVR), with a significant reduction in anxiety (71).

Patients with CTS are at increased risk of depression, particularly those with neuropathic pain (72). When psychological dysfunction is present, patients can present with symptoms similar to those with more advanced stages of physical/mechanical nerve dysfunction (determined by electrophysiologic testing) (72). Alsharif et al. found that carpal tunnel patients had similar rates of anger compared to controls, but significantly higher levels of anxiety and depression (73). Patients exhibiting higher neurotic symptoms had more negative CTS symptoms and decreased functional status (73). A multivariate controlled study of 732 patients showed that anxiety, depression, and worse EQ-5D scores (a measurement of quality of life) were significantly correlated with patient-reported symptom severity even after adjusting for age, sex, ethnicity, duration of CTS, smoking, alcohol, employment status, body mass index, and additional comorbidities (74).

A study assessing psychological phenotypes in 32 healthy controls and 108 CTS patients with either mild, moderate/severe, or no neuropathic pain found no significant difference between any of the groups on the Depression Anxiety and Positive Outlook Scale (DAPOS) (75). However, on the Pain Catastrophizing Scale (PCS), CTS patients showed significant increases in rumination and helplessness compared to healthy controls. CTS patients also experienced significantly elevated levels of escape, impaired cognition, and overall pain and anxiety (*p* = <0.0001) on the Pain Anxiety Symptoms Scale (PASS) compared to healthy controls (75). Comparecd to CTS with mild pain, CTS with moderate to severe neuropathic pain was associated with significantly higher overall PASS scores, as well as increased impaired cognition and escape behaviours (75). CTS patients with moderate-severe neuropathic pain also had significantly higher overall pain catastrophizing scores, as well as helplessness and rumination (75).

Patient outlook regarding symptoms and treatment may be a valuable predictor of outcomes in CTS patients (76). Sun et al. found that positive expectations and high patient comprehension of illness were independent associated with improved outcomes (76). In a study of 674 patients with CTS scheduled for surgery, increased self-reported CTS severity was associated with psychological distress, pain catastrophizing, consequences, identity, and emotional representation (77). After adjusting for patient baseline characteristics and comorbidities, these factors accounted for 20%–25% of variance in self-reported CTS severity (77). A prospective study with 307 patients found that only 58% of patients expected great releif or improvement prior to undergoing carpal tunnel release (CTR) surgery (78). Multivariate analysis indicated that “male sex, lower social deprivation, and lower BMI” were correlated with more positive expectations for surgery (78).

In 2005, Hobby et al. found a significant correlation between “psychological disturbance and pre-operative symptoms and disability”. Albeit, at 6 months post-operation, there was no link between these two factors (79). A more recent study, however, suggested that psychiatric evaluation may be helpful in determining candidacy for CTR, as their data showed a strong correlation between pre-existing mental illness and poor functional outcomes (80). Another study assessed pain anxiety and depression levels pre- and post-CTR, and reported that depression and anxiety was significantly correlated with symptom severity at baseline and 3 months post-surgery (81). In a study comparing psychological evaluation and electrodiagnostic testing as predictors of limb disability in CTS, psychological factors were highly correlated with increased disability (measured via QuickDash), while electrodiagnostic methods were not significantly associated with limb disability (80).

#### Cubital tunnel syndrome

Cubital tunnel syndrome (CuTS) results from entrapment of the ulnar nerve and can be caused by injuries to the elbow joint, excessive pressure, or excess stretching forces. While the pathophysiology is not always clear, studies have linked psychologic disturbances to incidence and severity of CuTS (82). In a study of 246 patients with CuTS, 17.9% had depression and 14.2% reported anxiety (83). Results showed a significant association between reported anxiety and higher severity levels (using the modified McGowan grade). Of note, no correlation was found between severity of symptoms and employment status or education (83).

#### Complex regional pain syndrome (CRPS)

CRPS is a debilitating syndrome that predominately affects the extremities and manifests in two forms. CRPS Type 1 presents with neuropathic pain without direct nerve damage, while Type 2 is caused by direct nerve trauma. Over 90% of individuals with CRPS have Type 1 (84). CRPS can present in children, adolescents, and adults (84). Females are more likely to develop CRPS, and the mean age of onset is between 40 and 50 years (85, 86).

It is estimated that around 50%–67% of CPRS patients have concurrent psychiatric illnesses (87).

A study of 39 CRPS patients found that 29 (74.4%) were at high risk of suicidal ideation, 19 (48.7%) were diagnosed with Major Depressive Disorder, and 7 (17.9%) were diagnosed with obsessive compulsive personality disorder (OCPD) (88). Specific factors associated with increased risk of suicidal ideation included depression, high severity of pain, and poor scores on the Global Assessment of Functioning Scale (a measure of mental illness severity) (88).

A study comparing patients with CPRS, major depressive disorders, and healthy controls showed that CPRS patients were significantly more likely to have hypochondria, depression, hysteria, paranoia, and psychasthenia (89). Further, there was a significant association between negative psychosocial variables and increased pain severity in CRPS patients (89). In pediatric populations, studies have shown that children with CRPS have lower levels of anxiety and depression compared to a control group with abdominal pain (90). Another study evaluating 101 children with CRPS also found that they had psychological profiles similar to children with other painful conditions (91). Lee et al. found that physical therapy in conjunction with at least one cognitive behavioral therapy session in children and adolescents led to improved pain and function at the end of 6 weeks, with sustained improvement at one year follow up (92).

Lohnberg et al. conducted a systematic review which revealed CRPS was linked to higher neurotic symptoms as well as a decrease in quality of life (93). Other reviews have concluded that the literature does not support a psychological predisposition to develop CRPS (94). Posttraumatic stress disorder (PTSD) has also been associated with an increased incidence of CRPS, with one study reporting that 58 out of 152 CRPS patients fit the criteria for PTSD, 50 of which showed PTSD symptoms prior to CRPS onset (95).

Bruehl et al. postulated that anxiety, depression, and life events may be involved with the development of CRPS through interactions with alpha adrenergic receptors (96). Some studies have argued against pre-existing psychological conditions as inciting factors of CRPS, and instead point to life events such as divorce or death of a family member as potential contributors to onset of CRPS (97). A 1992 study found that pre-existing mental illness had no significant effect on onset or development of CRPS (98). Another study showed similar amounts of anxiety and depression in CPRS patients compared to other diseases (87). Despite the lack of consensus on psychogenic origins of CRPS, it has been shown that psychological distress (identified after CRPS diagnosis) influences pain intensity and disease progression (99).

A last resort treatment to therapy resistant CRPS type 1 is amputation of the affected extremity. Schrier et al. found 4 factors affecting outcomes in these patients post amputation - low resilience, inadequate social support, psychological distress, and involvement in litigation in prior to amputation (100). These risk factors were associated with poor mobility and elevated levels of pain. Reoccurrence of pain was associated with psychiatric distress or involvement in litigation (100).

Singh et al. conducted a study of 12 patients with CRPS Type 1 to evaluate the effects of an interdisciplinary approach to CRPS treatment. They found that a treatment regimen including physical therapy, occupational therapy, water therapy, and psychotherapy can improve patient outcomes (101). At 2-year follow up, 66% of patients said the treatment was significantly helpful, and 75% were able to find employment after leaving their previous job due to CRPS (101).

Bruehl et al. suggested a multifactorial approach to CRPS including patient and family education, cognitive behavioral therapy, relaxation techniques, and psychiatric evaluation, in conjunction with typical treatment methods such as physical and occupational therapy (102). Others have recommended an interdisciplinary care team for CRPS patients which may include a pain psychologist/psychiatrist or a consultation-liaison psychiatrists specializing in mental management of comorbid conditions (103, 104).

## Discussion

Direct consequences of PNIs include motor and sensory deficits as well as neuropathic pain (9). These can lead to impaired quality of life and negative social impacts both in the workplace and in other daily activities. Recently, psychosocial factors have come under consideration as a potentially modifiable factors affecting the prognosis of nerve injury and recovery (105). Studies have shown PNIs to be associated with high levels of posttraumatic psychological distress, along with increased levels of depression, anxiety, personality disorders, and negative mental factors; all of which can lead to negative impacts on functional outcomes (17, 26). These symptoms can persist months after injury (26). If possible, negative impacts on quality of life may be mitigated by return to pre-injury activities (with or without accomodations) (106–112).

Our review of the literature shows a clear connection between psychosocial variables and symptom severity and progression in many acute and chronic PNIs. While there is little consensus on the causative role of psychological factors in peripheral neuropathy, outcomes of PNI appear to be at least modulated by these factors. In conditions known to have a strong connection to psychologic disturbance (e.g., Sciatica, CTS, and CRPS), psychological evaluation may help guide a comprehensive treatment plan (95). In traumatic PNIs, physicians should consider some form of pshycological screening and/or evaluation by a psychiatrist when quantitative assessment does not align with patients’ self-reported symptom severity and quality of life. Psychotherapy of some kind may be a valuable adjunct to traditional treatment modalities in these patients, and could ultimately lead to improved pain and functional outcomes, as well as quality of life.

## Data Availability

The original contributions presented in the study are included in the article/Supplementary Material, further inquiries can be directed to the corresponding author.
